# Brains in space: the importance of understanding the impact of long-duration spaceflight on spatial cognition and its neural circuitry

**DOI:** 10.1007/s10339-021-01050-5

**Published:** 2021-08-18

**Authors:** Alexander C. Stahn, Simone Kühn

**Affiliations:** 1 Department of Psychiatry, Unit of Experimental Psychiatry, Perelman School of Medicine, University of Pennsylvania, 4233 Guardian Dr, 1016 Blockley Hall, Philadelphia, PA 19104 USA; 2 Charité – Universitätsmedizin Berlin, corporate member of Freie Universität Berlin and Humboldt-Universität zu Berlin, Institute of Physiology, Charitéplatz 1, 10117 Berlin, Germany; 3 Lise Meitner Group for Environmental Neuroscience, Max Planck Institute for Human Development, 14195 Berlin, Germany; 4 Department of Psychiatry and Psychotherapy, University Medical Center Hamburg-Eppendorf, 20246 Hamburg, Germany

**Keywords:** Spatial cognition, Hippocampus, Spaceflight, Extreme environments

## Abstract

Fifty years after the first humans stepped on the Moon, space faring nations have entered a new era of space exploration. NASA’s reference mission to Mars is expected to comprise 1100 days. Deep space exploratory class missions could even span decades. They will be the most challenging and dangerous expeditions in the history of human spaceflight and will expose crew members to unprecedented health and performance risks. The development of adverse cognitive or behavioral conditions and psychiatric disorders during those missions is considered a critical and unmitigated risk factor. Here, we argue that spatial cognition, i.e., the ability to encode representations about self-to-object relations and integrate this information into a spatial map of the environment, and their neural bases will be highly vulnerable during those expeditions. Empirical evidence from animal studies shows that social isolation, immobilization, and altered gravity can have profound effects on brain plasticity associated with spatial navigation. We provide examples from historic spaceflight missions, spaceflight analogs, and extreme environments suggesting that spatial cognition and its neural circuitry could be impaired during long-duration spaceflight, and identify recommendations and future steps to mitigate these risks.

## Moon, mars and beyond: pushing the limits of human performance

On July 20, 1969, the world held its breath when Neil Armstrong and Buzz Aldrin landed on the Moon and became the first humans to explore the lunar surface. Now about fifty years later, space faring civilizations such as China, Japan, Europe, and India have joined the US and Russia in a new era of space exploration that goes well beyond the Moon. Spaceflight will play an increasingly important role in accelerating technological developments and transfer, establishing gateways to foster deep space exploration, seeking extraterrestrial life, establishing lunar colonies, venturing into deep space, and sending humans to Mars. Private partnerships and entities are fueling this race. *SpaceX* has been the first private company to transport astronauts to orbit and the International Space Station (ISS). Reusable rockets and super heavy launch systems such as *Starship* are expected to reduce costs of interplanetary flight and accelerate the developments needed for the colonization of other planets. Similar approaches and technologies have been recently announced by the China Academy of Launch Vehicle Technology, coinciding with China’s announcement to send its first human crew to Mars in 2033 and establish a large-scale settlement on the *Red Planet*. No matter which nation will win the next phase of this new *Space Race*, it will push the limits of human health and performance.

Space is a naturally hostile environment characterized by reduced gravity levels and various environmental, operational, and psychological stressors (e.g., radiation, hyperpcapnia, little separation between rest and works schedules, social isolation and confinement). Future exploratory missions will be considerably longer than current standard ISS missions. NASA’s reference mission to Mars is expected to comprise 1100 days. Deep space exploratory class missions could even span decades. These expeditions are considered the most dangerous and difficult explorations in the history of human spaceflight, and will expose crew members to unprecedented health and performance risks. The development of adverse cognitive or behavioral conditions and psychiatric disorders during those missions is considered a critical and unmitigated risk factor (Slack et al. 2016).To ensure successful future human space exploration, the risks must be precisely identified. Tools need to be provided that foster the efficient their efficient monitoring and prediction of adverse effects of spaceflight on brain and cognitive performance. In addition, target-specific countermeasures have to be established that help mitigating neurobehavioral impairments that may put astronaut health and mission success at risk.

## Risk of adverse brain and cognitive changes in response to long-duration spaceflight

Neuroimaging findings have revealed structural brain changes in response to spaceflight, including an upward shift of the brain, redistribution of cerebrospinal fluid, ventricular volume decreases, and widespread decreases in gray matter volume (Jillings et al. 2020; Roberts et al. 2017; Van Ombergen et al. 2018, 2019). These findings seem to stand in contrast to data on cognitive performance, revealing only minor impairments in response to spaceflight (Strangman et al. 2014). Whether and to what extent these changes lead to operational impairments and adverse behavioral conditions is currently not well understood. Retrospective analyses investigating the relationships between changes in cognitive performance using NASA’s cognitive test battery *WinSCAT* (Kane et al. 2005) and whole-brain structural analyses are inconclusive. Higher total ventricular volume was associated with reduced accuracy on a symbol substitution task (“Code Substitution” test), and faster response speed in an n-back paradigm (“Running Memory Continuous Performance” test) (Roberts et al. 2019). Other findings from standard 6-month ISS missions have shown significant decrements in manual dexterity, dual-tasking, motion perception, and a considerable degradation of a virtual navigation, i.e., car driving task immediately after return from space (Moore et al. 2019). Whereas these effects are expected to be relatively short-lived, i.e., minutes up to several days, they are considered a substantial risk for exploratory space missions, which involve transitions between gravitational levels (Harm et al. 2015). Data from a 1-year mission using NASA’s *Cognition* battery (Basner et al. 2015), assessing the cognitive performance of ten neuropsychological tests, suggests that adverse cognitive effects can persist up to 6 months postflight (Garrett-Bakelman et al. 2019). According to a review of studies that were performed during space missions, current data do not support cognitive deficits in low Earth orbit (Strangman et al. 2014). However, because of different approaches, methodologies, study durations, and small sizes, the effects of spaceflight on cognitive functions remain to be determined. However, because of different approaches, methodologies, study durations, and small sizes, the effects of spaceflight on cognitive functions remain to be determined (Mammarella 2020). Together, these data raise several key questions: (1) Could more complex cognitive and operational tasks be more sensitive to changes of inflight performance? (2) Is the neural circuitry underlying these tasks affected by spaceflight? (3) What are the long-term consequences of spaceflight on these tasks?

## Need to monitor visuospatial abilities before, during and after spaceflight

Operational performance can be assessed by simulating spaceflight-related tasks such as using robotic arms to capture a transiting spacecraft or to control a spacecraft to maneuver it and dock it to another vehicle (Ivkovic et al. 2019; Johannes et al. 2017). Successful completion of the maneuvers relates to various cognitive domains, including but not limited to situational awareness, planning, decision-making, object orientation, mental rotation, visual processing, fine motor control, and visual motor integration (Wong et al. 2020). It can be hypothesized that complex tasks assessing the encoding, processing, storage, and retrieval of visuospatial information could be particularly vulnerable during spaceflight. An analysis of shuttle missions revealed that touchdown speed in 20% of orbiter landings was outside acceptable limits, some of which were associated with pilot-induced oscillations, i.e., increasing flight corrections in opposite directions (Moore et al. 2008). In 1997, piloting errors during control of the TORU (Teleoperated Mode of (spacecraft) Control) system resulted in the collision of the supply spacecraft Progress M-34 with the MIR station, damaging the *Spektr* module and a solar panel (Morgan 2013). Likewise, several telerobotic incidents occurred on ISS, including a collision between the *Canadarm2*, the shuttle payload door, an external antenna, and Canadarm in which the two robotic arms crossed within 1.5 m of each other (Moore et al. 2019).

**Fig. 1:**
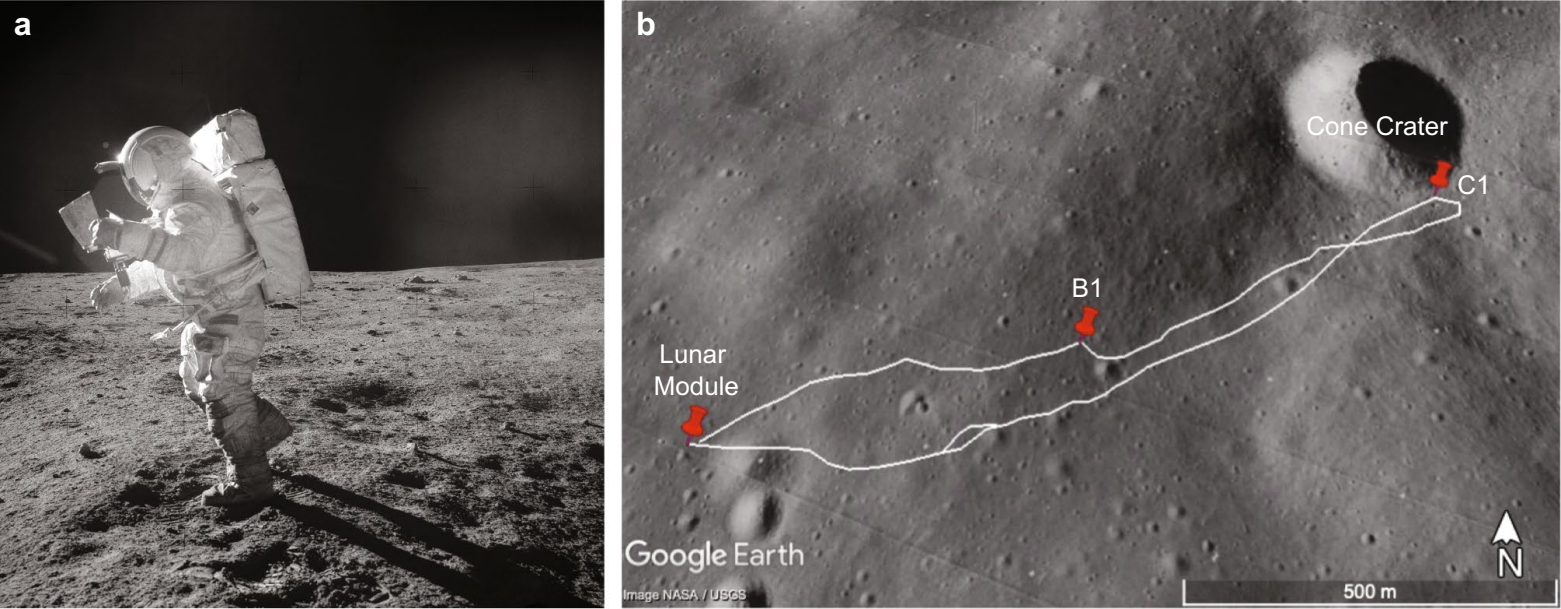
Traverse of Astronauts Edgar D. Mitchell and Alan Shepard during extravehicular activity (EVA-2) of the Apollo 14 mission. (**a**) Edgar D. Mitchell moves across the lunar surface as he is studying a map, trying to figure out where they are in vain; both astronauts thought they were much closer to Cone Crater than they actually were, and they did not recognize any landmarks in their view (picture was taken at location B1). (**b**) Outline of the traverse from Lunar Module to Cone Crater via B1 and back. Station C1 indicates “Saddle Rock,” where the last sample was retrieved before returning to the Lunar Module. Neither astronaut noticed that “Saddle Rock” was depicted as a landmark on their map. It was only after the completion of the mission that they had realized that were only 30 m from the rim of Cone Crater. Picture Credit: NASA/USGS and Google

Spatial updating, path integration, route learning, wayfinding, and cognitive mapping are also key to successfully navigating in small- and large-scale environments. The criticality of encoding representations about self-to-object relations and integrating this information into a spatial map of the environment for spaceflight operations was highlighted during the Apollo 14 mission. Astronauts Ed Mitchell and Alan Shepard had to walk to a crater located within a mile from their landing module. Having nearly reached the target destination, they had to abort the assignment because of spatial disorientation (Fig. 1). The difficulties associated with spatial navigation on the lunar surface were confirmed during the post-mission debriefs when Alan Shepard explained, “I felt that we had a navigation problem on EVA-2. I don't know why we didn't worry a little bit more about that pre-flight (…) Second, there’s no questions that it is easy to misjudge distances, not only above the surface [that is during the landing or from the Lunar Module windows] (…) but also distances along the surface.” (Heiken and Jones 2007).

**Fig. 2:**
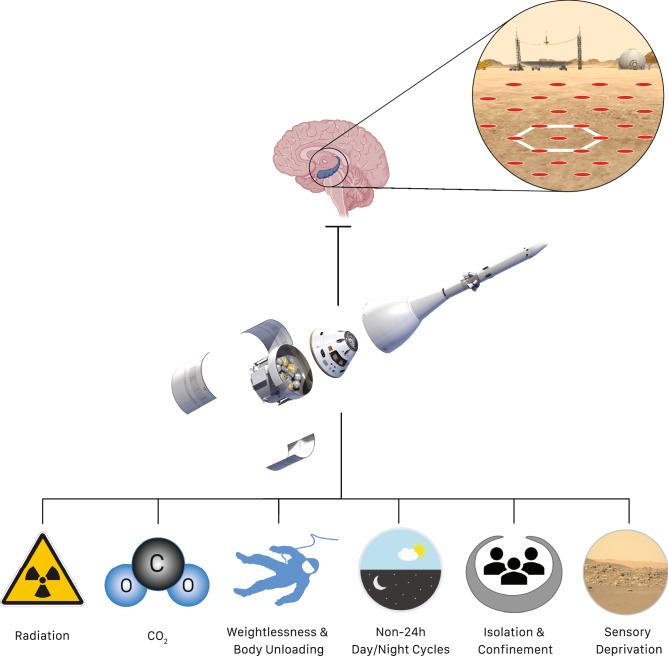
Environmental, operational, and psychological stressors associated with spaceflight. Ionizing radiation, hypercapnia (increased CO_2_ levels), altered vestibular stimulation and reduced physical activity in response to weightlessness, circadian disruptions and poor sleep due to altered day and night cycles, isolation and confinement, and sensory deprivation can have adverse effects on hippocampal plasticity. The hippocampus is critical for declarative memory formation, emotion processing, and spatial cognition. Together with the entorhinal cortex, the hippocampus supports the encoding, consolidation, and retrieval of spatial information by processing information about location (place cells of the hippocampus) relative to a grid map characterized by a hexagonal pattern (grid cells in the entorhinal cortex). Picture Credit: Schematic brain and hippocampus were created with BioRender.com. Spacecraft (middle) and right icon in bottom row (sensory deprivation): NASA; astronaut silhouette (third icon, bottom row) by Natasha Sinegina/CC BY); icon depicting Non-24h Day/Night cycles by icon-library.com

Finding your way in a new territory has always been a significant challenge for explorers. From an evolutionary perspective, the “(…) ability to estimate one’s own position and track and plan one’s own path in physical space is key to survival” (*Focus on Spatial Cognition* 2017). To identify the time course of visuospatial abilities in response to spaceflight, we have developed a specific battery of tasks that was recently flight-certified for use on the ISS. This battery assesses visuospatial memory formation, topographic mapping, path integration, and spatial updating. The battery has been tested in various spaceflight analogs on Earth. Experiments performed during parabolic flight maneuvers have shown that spatial updating is sensitive to gravitational changes, including micro- and hyper-gravity (Stahn et al. 2020). Recently, *Spatial Cognition* was selected as part of NASA’s CIPHER^1^ project. *Spatial Cognition* will investigate visuospatial changes and their neural basis in a total of 30 astronauts, equally assigned to 2-month, 6-month, and 1-year missions. Likewise, it will be essential to identify cortical and subcortical brain areas associated with the spatial encoding of landmark identities, retrieving spatial information, and processing visual features important for landmark recognition such as the hippocampal formation, striatum, precuneus, retrosplenial complex, parahippocampal place area, and the occipital place area (Epstein et al. 2017; Geerts et al. 2020; Hartley et al. 2014; Wolbers et al. 2008). The hippocampus is considered the human “inner” GPS by providing information about location (place cells of the hippocampus) relative to a grid map characterized by a hexagonal pattern generated by grid cell firing activity in the entorhinal cortex (Moser et al. 2015). Together, the hippocampus and entorhinal cortex play a critical role in exploring unfamiliar terrains, navigating on new planets, and performing complex operational visuospatial tasks (Fig. 2). To this aim, *Spatial Cognition* will combine behavioral data with multi-modal neuroimaging that are expected to provide new knowledge on the dose–response relationships between the length of spaceflight missions, brain changes, and their implications for spatial orientation and navigation. In addition, data will be collected up to a year after return from space to identify the time course of recovery.

## Environmental stressors, hippocampal plasticity and spatial cognition

The hippocampus, known as a highly plastic brain region that is key to complex spatial navigation, is vulnerable to various stressors associated with spaceflight, including but not limited to radiation, hypercapnia, altered vestibular stimulation, reduced physical activity levels, circadian disorders and poor sleep, sensory deprivation, and social isolation and confinement (Fig. 2). It is also possible that these stressors interact with each other. The strength and direction of such effects is currently not well understand, and also deserves further research.

### Radiation

Cosmic radiation is expected to be a critical risk for adverse neurobehavioral effects during spaceflight. Recent reviews reported considerable structural and functional damage of the brain that is particularly prominent in the prefrontal cortex and hippocampus, and associated with a broad range of adverse behavioral conditions, including taste aversion, reversal learning deficits, disrupted reinforcement behavior, contextual fear conditioning, and spatial learning and memory formation (Kiffer et al. 2019).

### Carbon dioxide

The limited capacity of air recycling systems on spacecraft can increase carbon dioxide (CO_2_) levels and lead to hypoxia/hypercapnia. Typical concentrations of CO_2_ on ISS range between 2 and 4 mmHg and can be up to ten-fold higher than on Earth (outdoors about 0.3 mmHg and in well-ventilated rooms about 0.5 mmHg). Animal studies have shown that chronic exposure to 0.3% CO_2_ concentrations can impair brain plasticity and behavior during early development (Kiray et al. 2014). In contrast to earlier reports (Satish et al. 2012), recent data found no or little change of varying levels of CO_2_ acutely on cognitive performance (Basner et al. 2021; Lee et al. 2019; Scully et al. 2019). The chronic effects of heightened CO_2_ levels on complex visuospatial abilities remain to be determined.

### Weightlessness

Weightlessness also affects the vestibular system, which goes beyond maintaining gaze and postural stabilization. Acute exposure to weightlessness and transitions between gravity levels significantly challenge the integration of neuro-vestibular signaling, associated with motion sickness and alterations in spatial abilities and sensorimotor functioning (Reschke and Clément 2018). Otoliths, graviceptors in the inner ear, rely on information from linear acceleration, and therefore cannot respond to tilt when gravity is lacking. Furthermore, projections of the vestibular pathways to the limbic system and neocortex play a critical role for brain plasticity, including spatial learning and memory formation (Smith 2017). Peripheral lesions of the vestibular pathways have been linked to atrophy in the hippocampus and spatial memory impairments that are long-lasting and may even be permanent (Smith et al. 2010).

### Body unloading

The lack of gravity also diminishes physical activity, known as a critical driver for brain plasticity and cognition (Voss et al. 2013). We recently showed that long-duration bed rest (> 1 month) has detrimental effects on various cognitive processes, including episodic memory formation in the hippocampus and parahippocampus (Brauns et al. 2019, 2021; Friedl-Werner et al. 2020). We also found that prolonged physical inactivity associated with bed rest induces circadian disruptions (Mendt et al. 2021a, b).

### Non-24 h light–dark cycles

Spaceflight is associated with circadian disruptions and adverse sleep (Barger et al. 2014; Flynn-Evans et al. 2016). A recent analysis of astronauts on standard ISS missions (average stay of 155 days) reported circadian misalignment in 20% of flight days and corresponding sleep losses of one hour per night (Flynn-Evans et al. 2016). Poor sleep is associated with neurodegenerative and neuropsychiatric conditions and hippocampal atrophy (Fjell et al. 2020). Chronic sleep deprivation has also been shown to lead to hippocampal atrophy across the adult lifespan (Fjell et al. 2020). The detrimental effects of sleep on the hippocampus are independent of stress hormones (Mueller et al. 2008), and structural hippocampal alterations have been observed after brief periods of sleep deprivation (Raven et al. 2019).

### Isolation, confinement, and sensory deprivation

Reduced sensory stimulation and sensory monotony experienced in isolated, confined, and extreme (ICE) environments are expected to be major contributors to adverse neurobehavioral conditions. Monotonous sensory stimulation, boredom, and isolation and confinement are severe stressors can lead to interpersonal tension and conflict, negative affect, work place errors, and increased mortality (Eastwood et al. 2012). For more than 30 years, space agencies have been investigating the effects of isolation and confinement using facilities designed to simulate spaceflight missions. These “laboratory” studies are characterized by highly controlled settings and have been referred to as isolated and controlled confinement (ICC) (Choukér and Stahn 2020). With few exceptions such as the Russian Mars500 study or the more recent SIRIUS projects using the NEK facility at the Institute of Biomedical Problems (IBMP) in Moscow, these studies are typically limited to short durations (< 60 days). Laboratory experiments also lack the complexity, unpredictability and risks associated with actual expeditions in extreme environments. Exploration expeditions bear the potential to study the effects of prolonged isolation and confinement in natural extreme environments and have been become known as isolated confined and extreme environments (ICE). The first reported data on the behavioral effects of isolation and confinement date back to Polar explorers, providing anecdotal evidence of the psychological and physiological challenges associated with long-duration Antarctic expeditions (Palinkas and Suedfeld 2008). Previous research in ICCs and ICEs focused on mood disorders, asthenia, psychosomatic reactions, psychosocial adaptations, and psychiatric emergencies (Mcphee and Charles 2009). To what extent social isolation directly causes brain changes, and cognitive performance impairments is less clear. Animal studies have shown that stress and social isolation disrupt hippocampal neurogenesis (e.g., Cinini et al. 2014; Gould et al. 1997; Schloesser et al. 2010), prevents exercise-induced hippocampal neurogenesis (Leasure and Decker 2009; Pereda-Pérez et al. 2013; Stranahan et al. 2006), selectively reduces hippocampal brain-derived neurotrophic factor (Scaccianoce et al. 2006), and impairs hippocampal long-term potentiation (Kamal et al. 2014).

To investigate whether similar effects can be detected in humans, we recently investigated the neurobehavioral responses to prolonged isolation associated with Antarctic overwintering (Stahn et al. 2019). T_1_- and T_2_-weighted magnetic resonance imaging (MRI) data were collected before and 1.5 months after a 14-month expedition to Antarctica to assess structural brain changes and compare these data to a control group matched for sex, age, and educational background. Hippocampal subfield volumes decreased after the expedition, namely bilateral dentate gyrus volume was significantly smaller in the expeditioners than in the control group (mean group decrease in volume ± SE: 32 ± 13 mm^3^, equivalent to a 7.2 ± 3% volume reduction). Whole-brain analyses using voxel-based morphometry (VBM) revealed further decreases of gray matter probability in the left parahippocampus (mean group decrease ± SE: 3.84 ± 0.72%), and in the right lateral and left medial and right lateral prefrontal cortex (PFC) (mean group decrease ± SE: 3.33 ± 0.48%; left medial PFC: mean group decrease ± SE: 2.99 ± 0.25%). Brain-derived trophic factor (BDNF), a protein key to brain plasticity and learning and memory formation (Egan et al. 2003; Harward et al. 2016), was determined in serum blood samples collected before, ten times during, and once after the expedition. After the first quarter of the expedition, serum BDNF concentration was reduced compared to the baseline measurement before the expedition and did not recover at 1.5 months after the end of the expedition (mean reduction ± SE: 11 ± 1.5 ng/mL, 45 ± 4.9%). Reductions in BDNF from pre- to post-mission were associated with decreases in dentate gyrus volume (*R*^2^ = 0.47). The reductions in dentate gyrus volume were also associated with lower cognitive performance in tests of spatial processing (*R*^2^ = 0.87) and the resolution of response conflict (*R*^2^ = 0.82), but there was no reduction in performance in other cognitive tests (i.e., Digit Symbol Substitution, Stroop Congruent task).

## Need for target-specific countermeasures

To mitigate adverse neurobehavioral effects of prolonged spaceflight on spatial cognition and its neural basis, target-specific countermeasure will be needed that go beyond current practices such as exercise, lower body negative pressure, and nutritional supplementation. For instance, specific types of video gaming have the potential to enhance brain plasticity (Kühn et al. 2014a, b; 2017). Further, specific training programs aimed at improving operational performance skills (e.g., Johannes et al. 2017) can be expected to improve visuospatial abilities and affect their neural circuitry. Moreover, combining physical activity with virtual environments could be promising (Vessel and Russo 2015). Preliminary data analyses of the NASA sponsored project *Hybrid Training* showed that voluntary exercise on bicycle ergometer combined with a visual sensory stimulation could mitigate some of the neurobehavioral effects in response to 14 months of isolation and confinement associated with overwintering at Neumayer III station in Antarctica. These effects were manifested as increased BDNF concentrations and reductions in hippocampal subfield volume and whole-brain gray matter and support the role of physical activity as a key driver of brain plasticity (Vivar and van Praag 2017).

**Fig. 3:**
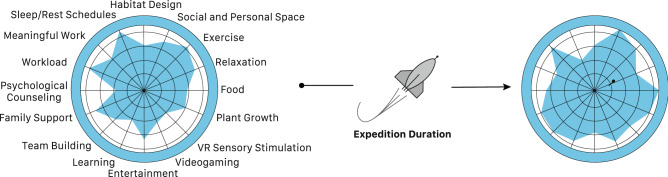
Individualized countermeasures (ICount) to mitigate adverse neurobehavioral effects. To mitigate the neurobehavioral risks associated with long-duration spaceflight, a comprehensive “toolbox” of countermeasures is needed. The figure lists some examples that will play a critical role in reducing the risks of adverse cognitive and behavioral effects and psychiatric disorders during long-duration expeditions. Some of the countermeasures listed such as exercise, videogaming, diet and nutritional supplementation, sleep hygiene, and self-adapted visuo-spatial learning tasks will also help to maintain hippocampal plasticity and spatial cognition. The relationships between individual countermeasures will vary between and within individuals. The relative importance of specific strategies will vary during the course of mission, requiring a constant reevaluation of the crewmembers’ individual needs. Note that the figure is a schematic illustration, and the weights of the interventions are used to reflect their dynamic nature through an expedition, but do not suggest any importance of one countermeasure over the other

Given the range of environmental, operational and psychological conditions and stressors, it is expected that there is no single countermeasure that will serve as a universal remedy. In addition, it is possible that the responses to the countermeasures will vary between individuals. Countermeasure must therefore be understood as a dynamic construct that is optimized relative to the individual needs as a function of mission duration. The concept of individualized countermeasure that we like to term as “ICount” is summarized in Fig. 3. A multiplicity of methodologies and approaches could be combined in a toolbox that is flexibly adapted to address the crews’ individual needs. The countermeasures range from habitat design (e.g., lighting, personal and social space), to exercise, workload considerations including variations in meaningful work, sleep/rest schedules, relaxation techniques, videogaming, entertainment, virtual reality, plants, food, and other sensory augmentation measures, to strategies for maintaining and enhancing crew cohesion, psychological counseling and family support. The variety of approaches will be critical to maximize the stimulation and their synergies, and consider phenotypic differences, and the dynamic nature of individual preferences for specific needs during long-duration expeditions.

## Summary and Conclusions

Outer space is considered the most extreme environment for human mankind. Without support systems and protective suits, life in space or on other planets in our solar system is not possible. Even prolonged stays in the habitat of a spacecraft pose significant physiological and psychological challenges. Spaceflight affects every organ system. In addition to microgravity the spacecraft setting is characterized by multiple environmental toxicants and operational stressors such as radiation, noise, hypercapnia, hypoxia, decompression, dietary restrictions, fluid shifts, increased intracranial pressure, non-24 h light–dark cycles, acute operational shifts in sleep timing, psychological factors related to high workload under pressure, operational and interpersonal distress, and isolation and confinement. The effects of space travel on brain and behavior are currently not well understood but are considered a high and unmitigated risk for future long-duration space missions.﻿

Studies in animals and ground-based spaceflight analogs suggest that the spatial cognition and its neural basis could be particularly vulnerable to future long-duration space missions. Future studies are therefore critically needed to (1) understand the effects of extreme environments and spaceflight on spatial cognition and its neural circuitry, (2) demonstrate and verify the techniques needed to monitor, diagnose, and prevent such effects, and (3) develop target-specific countermeasures to mitigate adverse effects on visuospatial abilities. In addition, imaging and cognitive data should be complemented by biochemical assessments, and advances in multi-omics technologies such as genomics, transcriptomics, proteomics, and metabolomics. They will be critical to close knowledge gaps of the underlying molecular mechanisms and genetic drivers of neurobehavioral adaptations in extreme environments.﻿ Combining brain imaging, cognitive and biochemical methodologies, and outcomes could provide the basis to better understand and characterize the type, extent, cause, and mechanisms of adverse neurobehavioral effects and their phenotypic signatures.﻿

The integration of imaging, physiological, biochemical, and behavioral data will contribute to the space agencies’ goal to provide knowledge, technologies, and tools to enable safe, reliable, and productive human space exploration. At the same time, they can also benefit research and applications on Earth. Spaceflight analogs such as isolation experiments, Antarctic expeditions, and bed rest studies, can provide unique standardized settings that induce neurophysiological and psychological conditions that typically evolve over long time spans, and cannot be replicated in typical laboratory settings. The opportunity to study prospectively the time course of brain and behavioral changes in time lapse in healthy adults, characterize them before any clinical manifestations occur, and follow them up through recovery can help understand the effects and the biological basis of aging-related cognitive decline, social isolation, clinical manifestations associated with impaired physical mobility, or lifestyle changes in response to pandemics.

## Footnotes

1 CIPHER stands for Complement of Integrated Protocols for Human Exploration Research, and is a project comprising 17 investigators that integrate various disciplines to investigate multiple physiological, biological, and psychological aspects to short-duration (2-months), standard-duration (6-months), and long-duration (12-months) human spaceflight missions.
